# Concurrent Modified Constant Modulus Algorithm and Decision Directed Scheme With Barzilai-Borwein Method

**DOI:** 10.3389/fnbot.2021.699221

**Published:** 2021-06-10

**Authors:** Tongtong Xu, Zheng Xiang, Hua Yang, Yun Chen, Jun Luo, Yutao Zhang

**Affiliations:** ^1^Department of Telecommunication Engineering, Xidian University, Xi'an, China; ^2^Guangdong Shenglu Telecommunication Tech. Co., Ltd, Foshan, China

**Keywords:** robot technology, Barzilai-Borwein method, decision-directed, blind equalization, modified constant modulus algorithm

## Abstract

At present, in robot technology, remote control of robot is realized by wireless communication technology, and data anti-interference in wireless channel becomes a very important part. Any wireless communication system has an inherent multi-path propagation problem, which leads to the expansion of generated symbols on a time scale, resulting in symbol overlap and Inter-symbol Interference (ISI). ISI in the signal must be removed and the signal restores to its original state at the time of transmission or becomes as close to it as possible. Blind equalization is a popular equalization method for recovering transmitted symbols of superimposed noise without any pilot signal. In this work, we propose a concurrent modified constant modulus algorithm (MCMA) and the decision-directed scheme (DDS) with the Barzilai-Borwein (BB) method for the purpose of blind equalization of wireless communications systems (WCS). The BB method, which is two-step gradient method, has been widely employed to solve multidimensional unconstrained optimization problems. Considering the similarity of equalization process and optimization process, the proposed algorithm combines existing blind equalization algorithm and Barzilai-Borwein method, and concurrently operates a MCMA equalizer and a DD equalizer. After that, it modifies the DD equalizer's step size (SS) by the BB method. Theoretical investigation was involved and it demonstrated rapid convergence and improved equalization performance of the proposed algorithm compared with the original one. Additionally, the simulation results were consistent with the proposed technique.

## Introduction

As is known, inter-symbol interference (ISI) is a major impairment for improvement in capacity or data transmission rate in wireless communication systems (WCS), especially in the field of robot wireless control (Lewis et al., 2020; Ye et al., 2020). Over the past decades, a variety of equalization methods, as well as channel estimation algorithms (Macchi and Eweda, 1984; Johnson et al., 1998; Yang et al., 2002; Chen and Chng, 2004; Ashmawy et al., 2009; Liyi et al., 2009; Yuan and Lin, 2010; Kim et al., 2012; Bhotto and Bajić, 2015; Haykin, 2015; Shah et al., 2017), have been proposed to relieve the ISI effects, thus compensating for channel distortion. The essence of equalization is to filter the signal, and the adaptive filter can automatically adjust its tap system, so as to achieve the best channel compensation effect. As the adjustment strategy of tap coefficient of adaptive filter, adaptive algorithm plays a decisive role in the filtering effect. In general, the adaptive equalizer needs to send a known training sequence to adjust the adaptive equalizer before sending signals, so as to achieve the best equalization effect. However, in the actual communication, the receiver cannot obtain any known information. Blind equalization (BE) is a method widely used for recovery of transmitted symbols of superimposed noise without providing the desired response externally (Yang et al., 2002). The constant modulus algorithm (CMA) (Johnson et al., 1998) is regarded as the most classical BE algorithm, in virtue of its simple mechanism, good stability, and high efficiency. The approach, however, takes longer to converge and barely achieve reasonable mean square error (MSE). The result obtained may not be able to ensure good system performance, which is a serious weakness of consideration.

Researchers have put forward a number of interesting solutions (Macchi and Eweda, 1984; Chen and Chng, 2004; Ashmawy et al., 2009; Liyi et al., 2009; Yuan and Lin, 2010; Kim et al., 2012; Bhotto and Bajić, 2015; Elsidig and Babiker, 2018) to this problem. Liyi et al. (2009) proposed a variable step size (VSS) technique and applied it to CMA for 16 quadrature amplitude modulation (QAM) signals. It improves the performance of the equalizer to some extent. However, it fails to take the higher-order QAM signals into account, which is another problem of this approach. On this basis, the modified constant modulus algorithm (MCMA) (Liyi et al., 2009) was proposed, which could improve the CMA performance by achieving low steady-state (SS) MSE. Also, joint carrier phase recovery is no longer needed. An alternative solution is switching to a decision directed (DD) mode, which does not require a threshold level or estimating the state of convergence and can minimize the SSMSE of remaining CMA (Macchi and Eweda, 1984). However, to make the transfer successful, the SSMSE of the CMA must be sufficiently low (Chen and Chng, 2004). In fact, CMA may not be able to achieve MSE as expected, at such a low level. Another possible method is to combine the existing blind equalization algorithm with the optimization algorithm to obtain improved equalization performance.

Generally, the optimization problem is to optimize the value of the variable *x* so as to minimize the objective function f(x). Non-linear optimization means that f(x) is essentially a non-linear one. There are many kinds of non-linear optimization algorithms, which can be divided into line search and trust region according to the different iterative methods, including the Newton method and the quasi Newton method, according to the different second-order approximation methods. For non-linear multidimensional unconstrained problems, gradient method is the simplest and the most basic. It has the advantages of simplicity and reliability, but its disadvantage is slow convergence. The selection of step size (SS) in gradient method has a great influence on the algorithm. After the Barzilai-Borwein (BB) method size is proposed, a new research upsurge of gradient method has arisen. Because there are many similarities between the optimization process of gradient method and the equalization process of blind equalization algorithm, the combination of the two methods can be considered to improve its performance based on the original equalization process.

In this study, we focus on the joint MCMA-DD equalization algorithm (Ashmawy et al., 2009) and propose a concurrent MCMA and DD with the Barzilai-Borwein (BB) method (Barzilai and Borwein, 1988) blind equalization algorithm for the WCSs using QAM signals. In the previous study (Macchi and Eweda, 1984), the authors have suggested concurrent operation of a DD equalizer and a CMA equalizer, instead of switching to a DD scheme after convergence of the CMA. The method is also applicable to MCMA. Concurrent MCMA and DD equalizers have significant improvements in equalization performance over MCMA. Based on the output error of blind equalizer (BE), the proposed method could adjust the SS of the weight with updating formula by using BB method. It could further enhance the convergence speed (CS), maintain the ISI and reduce the SSMSE.

## Blind Equalization

### System Model

For an adaptive blind channel equalization (BCE) system, the signal that is received for the interval of the kth symbol *x*(*k*) could be calculated by:

(1) $$\begin{array}{l} x\left(k\right)=\overset{L-1}{\underset{i\;=\;0}{\sum }}h\left(i\right)s\left(k-i\right)+v\left(k\right) \end{array}$$

where *h*(k) refers to the impulse response. The transmitted sequence *s*(*k*) is determined based on the QAM signal set. *v*(*k*) is taken as additive white Gaussian noise (AWGN) with mean of 0 and variance of σ^2^. Hence, the actual equalizer output may be written as

(2) $$\begin{array}{l} \overset{\wedge }{s\left(k\right)}=w^{T}\left(k\right)x\left(k\right) \end{array}$$

where $w\left(k\right)={\left[w_{0}\left(k\right),w_{1}\left(k\right),\cdots \;,w_{N-1}\left(k\right)\right]}^{T}$ refers to the weight vector (WV) of the BE and ***x***(*k*) = [*x*(*k*), *x*(*k* − 1), ⋯ , *x*(*k* − *N* + 1)]^*T*^ refers to the received sample vector.

### Concurrent MCMA and DD

The MCMA changes the cost function of CMA (Shah et al., 2017) from real field to complex field, and its cost function is:

(3) $$\begin{array}{l} \psi_{MCMA}\left(k\right)=\psi_{MCMA,R}\left(k\right)+\psi_{MCMA,I}\left(k\right) \end{array}$$

where ψ_MCMA,R_(*k*) and ψ_MCMA,I_(*k*) are the cost functions for real and imaginary parts, respectively.

The MCMA equalizer output $\overset{\wedge }{s\left(k\right)}$ could be calculated by

(4) $$\begin{array}{l} \overset{\wedge }{s\left(k\right)}=w_{MCMA}^{T}\left(k\right)x\left(k\right) \end{array}$$

The equalizer WV ***w***_*MCMA*_(*k*)can be updated by:

(5) $$\begin{array}{l} w_{MCMA}\left(k+1\right)=w_{MCMA}\left(k\right)-\mu_{MCMA}\nabla \psi_{MCMA}\left(k\right)\\ \;=w_{MCMA}\left(k\right)-\mu_{MCMA}e_{MCMA}\left(k\right)x^{*}\left(k\right) \end{array}$$

where μ_*MCMA*_ is the step size.

The error signal *e*_*MCMA*_(*k*) = *e*_*MCMA, R*_(*k*)+*je*_*MCMA, I*_(*k*) is given by

(6) $$\begin{array}{l} e_{MCMA,R}\left(k\right)=\overset{\wedge }{s_{R}\left(k\right)}\left({\left|\overset{\wedge }{s_{R}\left(k\right)}\right|}^{2}-R_{R}\right) \end{array}$$

(7) $$\begin{array}{l} e_{MCMA,I}\left(k\right)=\overset{\wedge }{s_{I}\left(k\right)}\left({\left|\overset{\wedge }{s_{I}\left(k\right)}\right|}^{2}-R_{I}\right) \end{array}$$

where R is a normal number.

**Figure 1:**
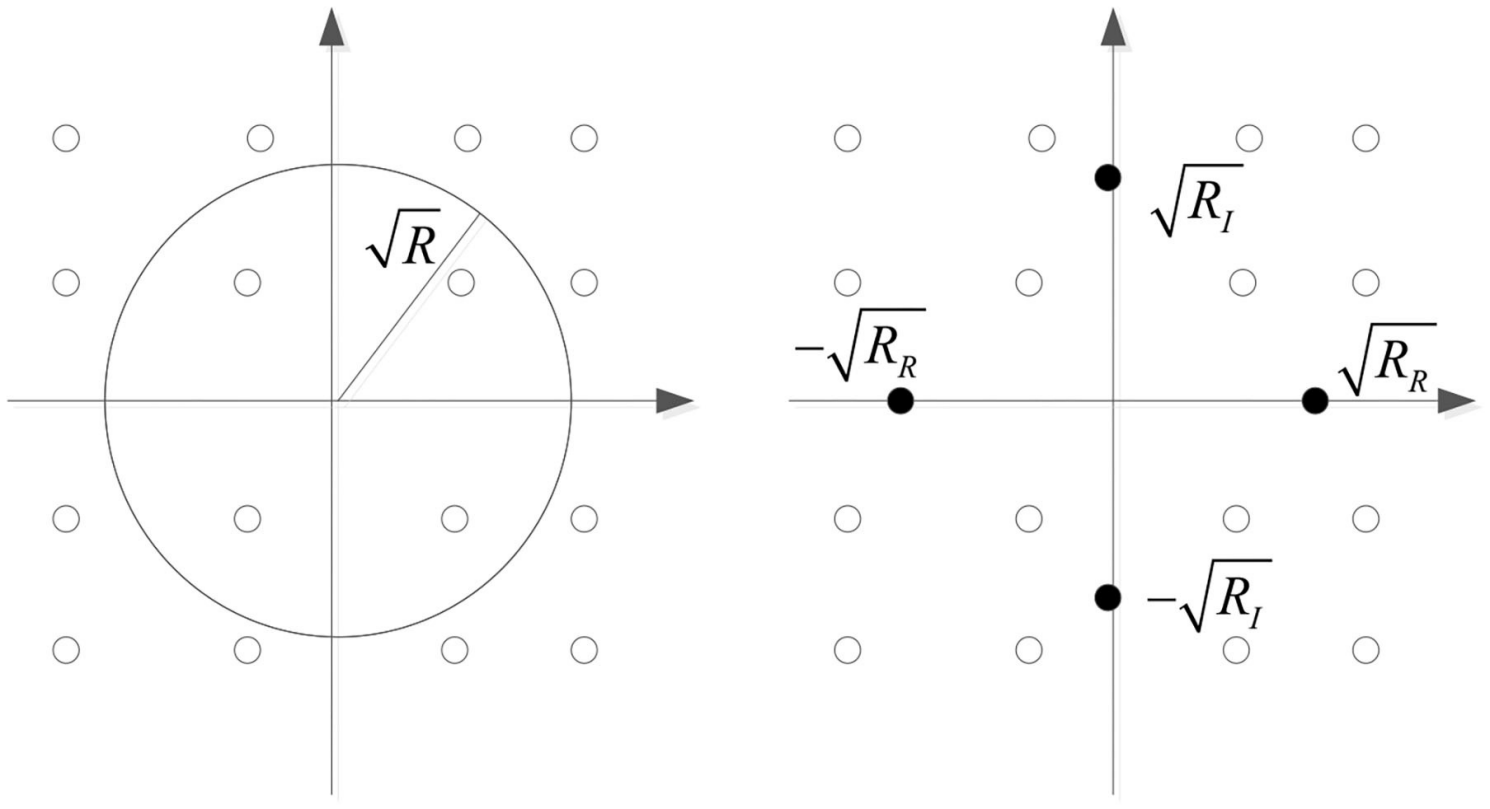
Error signal track of CMA and MCMA.

For CMA algorithm, when *e*(*k*) = 0, ${\left|\overset{\wedge }{s\left(k\right)}\right|}^{2}-R=0$, it is proved that the algorithm tends to make the equalized signal fall on the circle with $\sqrt{R}$ as the radius. In the MCMA algorithm, the real part and the imaginary part of the signal are handled separately. When *e*(*k*) = 0, ${\left|\overset{\wedge }{s_{R}\left(k\right)}\right|}^{2}-R_{R}=0$ and ${\left|\overset{\wedge }{s_{I}\left(k\right)}\right|}^{2}-R_{I}=0$ are both valid. After equalization, the real part of the signal will fall on the real axis $\pm \sqrt{R_{R}}$, and the imaginary part will attempt to fall on the virtual axis $\pm \sqrt{R_{I}}$. The error signal tracks of both are shown in Figure 1. After comparing the convergence of error signal *e*(*k*) between CMA and MCMA, it is not difficult to see that the MCMA comprehensively considers the amplitude and phase equalization, which can correct the phase deflection during transmission to a certain extent and achieve a better equalization effect. The concurrent MCMA and DD algorithm involves a MCMA equalizer and a DD equalizer, which concurrently operate. Explicitly, let ***w*** = ***w***_*MCMA*_ + ***w***_*DD*_, where ***w***_*DD*_ refers to the WV of the DD equalizer.

**Figure 2:**
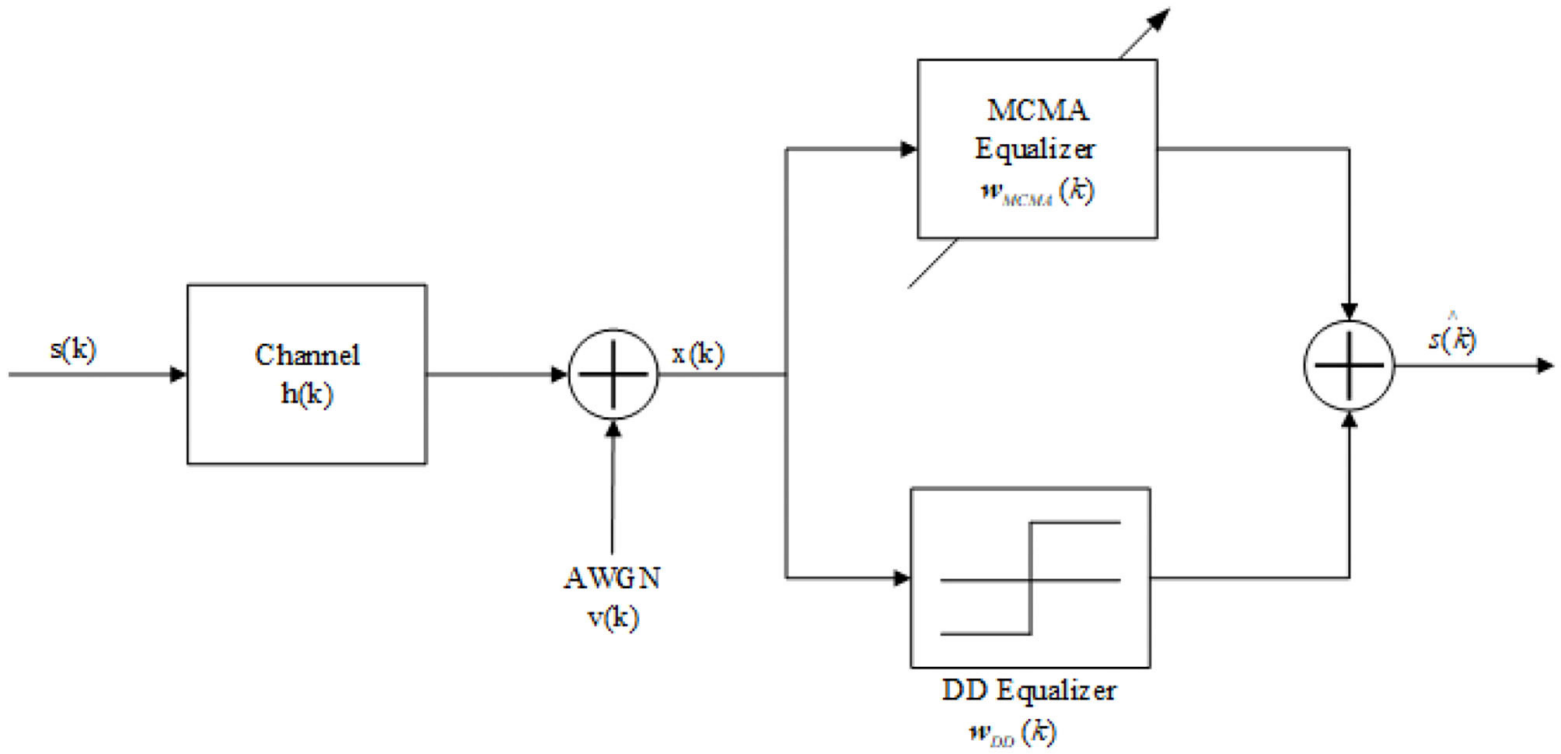
The baseband model of the concurrent MCMA and DD BCE system.

Figure 2 shows the baseband model of the concurrent MCMA and DD blind channel equalization system.

The cost function of the DD equalizer is:

(8) $$\begin{array}{l} \psi_{DD}\left(k\right)=E\left[{\left|\mathbb{Q}\left[\overset{\wedge }{s\left(k\right)}\right]-\overset{\wedge }{s\left(k\right)}\right|}^{2}\right] \end{array}$$

where $\mathbb{Q}\left[\overset{\wedge }{s\left(k\right)}\right]$ is the equalizer output of the quantized MCMA:

(9) $$\begin{array}{l} \mathbb{Q}\left[\overset{\wedge }{s\left(k\right)}\right]=\arg\underset{s\left(k\right)\in S}{\min}{\left|\overset{\wedge }{s\left(k\right)}-s\left(k\right)\right|}^{2} \end{array}$$

The DD adaptation is performed immediately after MCMA adaptation. The equalizer output could be calculated by:

(10) $$\begin{array}{l} \overset{\wedge }{s\left(k\right)}=w_{MCMA}^{T}\left(k+1\right)x\left(k\right)+w_{DD}^{T}\left(k\right)x\left(k\right) \end{array}$$

The DD equalizer updated coefficient is expressed as

(11) $$\begin{array}{l} w_{DD}\left(k+1\right)=w_{DD}\left(k\right)-\mu_{DD}\nabla \psi_{DD}\left(k\right)\\ \;=w_{DD}\left(k\right)-2\mu_{DD}e_{DD}\left(k\right)x^{*}\left(k\right) \end{array}$$

where μ_*DD*_ denotes the SS of the DD equalizer and $e_{DD}\left(k\right)=\overset{\wedge }{s\left(k\right)}-\mathbb{Q}\left[\left(\overset{\wedge }{s\left(k\right)}\right)\right]$ is the value of the estimated error.

It could be seen that the updated ***w***_*DD*_ occurs after the CMA equalization and before the equalizer hard decision. Compared to pure MCMA, concurrent MCMA and DD adaptation exhibits improved CS and reduced SSMSE. The μ_*DD*_ chosen can be significantly larger than μ_*MCMA*_.

## The Proposed Concurrent MCMA and DD With BB Method

### The BB Method

For multidimensional unconstrained optimization problems, i.e., $\underset{x\in {ℜ}_{n}}{\min}f\left(x\right)$, the gradient method is the simplest solution, which employs the negative gradient direction as the search direction. The iteration formula for this method is

(12) $$\begin{array}{l} x_{k+1}=x_{k}-\lambda_{k}g_{k} \end{array}$$

where λ_*k*_ is the iteration step and *g*_*k*_ = ∇*f*(*x*_*k*_) is the search direction.

The BB method, also known as the two-step gradient method, was initially proposed by Barzilai and Borwein (1988). It was applied to solve various unconstrained optimization problems (Dai et al., 2006; Nesterov, 2013; Tan et al., 2016) later. The method has an improved convergence rate compared with the gradient method. The proposal of Barzilai and Borwein SS factor has raised the upsurge of studying gradient method, and some new methods have also emerged (Li et al., 2019).

The fundamental concept of the BB method is to use the information of the current point and the previous point to determine the step factor and convert Equation (12) to

(13) $$\begin{array}{l} x_{k+1}=x_{k}-D_{k}g_{k} \end{array}$$

where *D*_*k*_ = λ_*k*_*I*, I is the unit matrix. In order to make *D*_*k*_ own “quasi-Newton” properties, calculate λ_*k*_ to satisfy the formula

(14) $$\begin{array}{l} \min{\left\|d_{k-1}-D_{k}{\tilde{g}}_{k-1}\right\|}_{2} \end{array}$$

or

(15) $$\begin{array}{l} \min{\left\|D_{k}^{\text{-}1}d_{k-1}-{\tilde{g}}_{k-1}\right\|}_{2} \end{array}$$

where *d*_*k*−1_ = *x*_*k*_ − *x*_*k*−1_ and ${\tilde{g}}_{k-1}=g_{k}-g_{k-1}$.

From Equations (13, 14), the formulas

(16) $$\begin{array}{l} \lambda_{k}=d_{k-1}^{T}{\tilde{g}}_{k-1}/{\left\|{\tilde{g}}_{k-1}\right\|}_{2}^{2} \end{array}$$

and

(17) $$\begin{array}{l} \lambda_{k}={\left\|d_{k-1}\right\|}_{2}^{2}/d_{k-1}^{T}{\tilde{g}}_{k-1} \end{array}$$

can be obtained.

Barzilai and Borwein have proved that the algorithm, whose SS is determined by Equations (16, 17), is linearly convergent in R domain, and the convergence order is $\sqrt{2}$. The result is true for almost initial values.

The algorithm flow of BB method is shown in Algorithm 1.

**Algorithm 1:** Barzilai and Borwein method.

1:	**Initialization**
2:	Initialize $x_{1}\in {ℜ}^{n}$, tolerance 0 < ε < 1,*k* = 1.
3:	**Loop Processing**
4:	*g*_*k*_ = ∇*f*(*x*_*k*_).
5:	if ‖*g*(*k*)‖_2_ ≤ ε
	the loop end.
6:	if *k* = 1
	get the iteration stepλ_*k*_by line search method (Nesterov, 2013).
	Or else,
	get the iteration stepλ_*k*_by Equations (16, 17.
7:	Calculate Equation (12) to get *x*_*k*+1_.
8:	*k* = *k*+1.
9:	Go to step 5.

### The Concurrent MCMA and DD With BB Method

We considered applying Equation (17) to update the value of μ_*DD*_ in concurrent MCMA and DD algorithm, which could improve the CS. Thus μ_*DD*_(*k*) may be expressed as

(18) $$\begin{array}{l} \mu_{DD}\left(k\right)={\left\|d\left(k\right)\right\|}_{2}^{2}/d^{T}\left(k\right)\tilde{g}\left(k\right) \end{array}$$

where

(19) $$\begin{array}{l} d\left(k\right)=x\left(k+1\right)-x\left(k\right) \end{array}$$

and

(20) $$\begin{array}{l} \tilde{g}\left(k\right)=\nabla \psi_{DD}\left(k+1\right)-\nabla \psi_{DD}\left(k\right)\\ \;=2e_{DD}\left(k+1\right)x^{*}\left(k+1\right)-2e_{DD}\left(k\right)x^{*}\left(k\right) \end{array}$$

Equation (11) can be changed to

(21) $$\begin{array}{l} w_{DD}\left(k+1\right)=w_{DD}\left(k\right)-\mu_{DD}\left(k\right)\nabla \psi_{DD}\left(k\right)\\ \;=w_{DD}\left(k\right)-2\mu_{DD}\left(k\right)e_{DD}\left(k\right)x^{*}\left(k\right) \end{array}$$

The entire BE process is summarized in Algorithm 2.

**Algorithm 2:** The proposed algorithm.

1:	**Initialization**
2:	Initialize ***w***_*MCMA*_ and ***w***_*DD*_ with zeros and substitute 1 for the central tap. Initialize μ_*MCMA*_ and μ_*DD*_.
3:	**Loop Processing**
4:	Calculate Equation (4) to get the output of MCMA equalizer.
5:	Update the weights vector ***w***_*MCMA*_ of MCMA equalizer using Equation (5).
6:	Calculate $\overset{\wedge }{s\left(k\right)}$ using Equation (10) to get the output of MCMA-DD equalizer.
7:	Update the weights vector ***w***_*DD*_ of DD equalizer using Equation (21).
8:	Update the step size μ_*DD*_ of DD equalizer using Equation (18) by BB method.
9:	Calculate MSE and ISI.
10:	**Until** convergence

From what has been discussed above, it is obvious that the value of μ_*DD*_ is mainly related to $\tilde{g}\left(k\right)$. MCMA has performed better in the initial stage of the equalization iteration and plays a leading role at this stage. The difference of the estimation error decreases gradually but the μ_*DD*_ gradually increases with the progress of the equalization iteration process, which makes DD take the dominant position. Hence, the convergence has been accelerated and the SSMSE has been further reduced.

## Performance Analysis and Simulation Results

Performance of the proposed concurrent MCMA and DD with BB algorithm was demonstrated by means of simulations. Generally, the performance of BE algorithm is mainly evaluated using convergence rate, average stability error, ISI residual, computational complexity, algorithm implementation difficulty and other indicators. Convergence rate, MSE and ISI are the three most important indicators. The CS is indeed closely related to the applicability of the real-time systems. MSE and ISI are closely related to the error correction ability after the algorithm converges, which directly affects its performance. Therefore, the equalizer should shorten the convergence time as much as possible and improve the CS without sacrificing the average error. The MSE was defined as

(22) $$\begin{array}{l} MSE=\frac{1}{N}\overset{N}{\underset{k\;=\;1}{\sum }}{\left|\mathbb{Q}\left[\overset{\wedge }{s\left(k\right)}\right]-\overset{\wedge }{s\left(k\right)}\right|}^{2} \end{array}$$

The residual ISI defined by

(23) $$\begin{array}{l} ISI=\frac{\overset{N}{\underset{k\;=\;1}{\sum }}\left(\left|h\left(k\right)*w^{*}\left(k\right)\right|\right)-{\left|h\left(k\right)*w^{*}\left(k\right)\right|}_{\max}}{{\left|h\left(k\right)*w^{*}\left(k\right)\right|}_{\max}} \end{array}$$

The performance simulations of MCMA, concurrent MCMA-DD and concurrent MCMA-DD with BB were executed in 30-dB SNR with 64-QAM. The channel impulse response was set to be *h* = [0.005, 0.009, −0.0024, 0.854, −0.218, 0.049, −0.016] T, and then the channel was normalized.

The SS of the MCMA had to be defined as $\mu_{MCMA}=2\times 10^{-6}$. The two step sizes of the concurrent MCMA-DD equalizer were defined to be $\mu_{MCMA}=2\times 10^{-6}$ and $\mu_{DD}=10^{-4}$, respectively. For the concurrent MCMA-DD with BB equalizer, the two step sizes were set to $\mu_{MCMA}=2\times 10^{-6}$ and the initial$\mu_{DD}=10^{-3}$.

**Figure 3:**
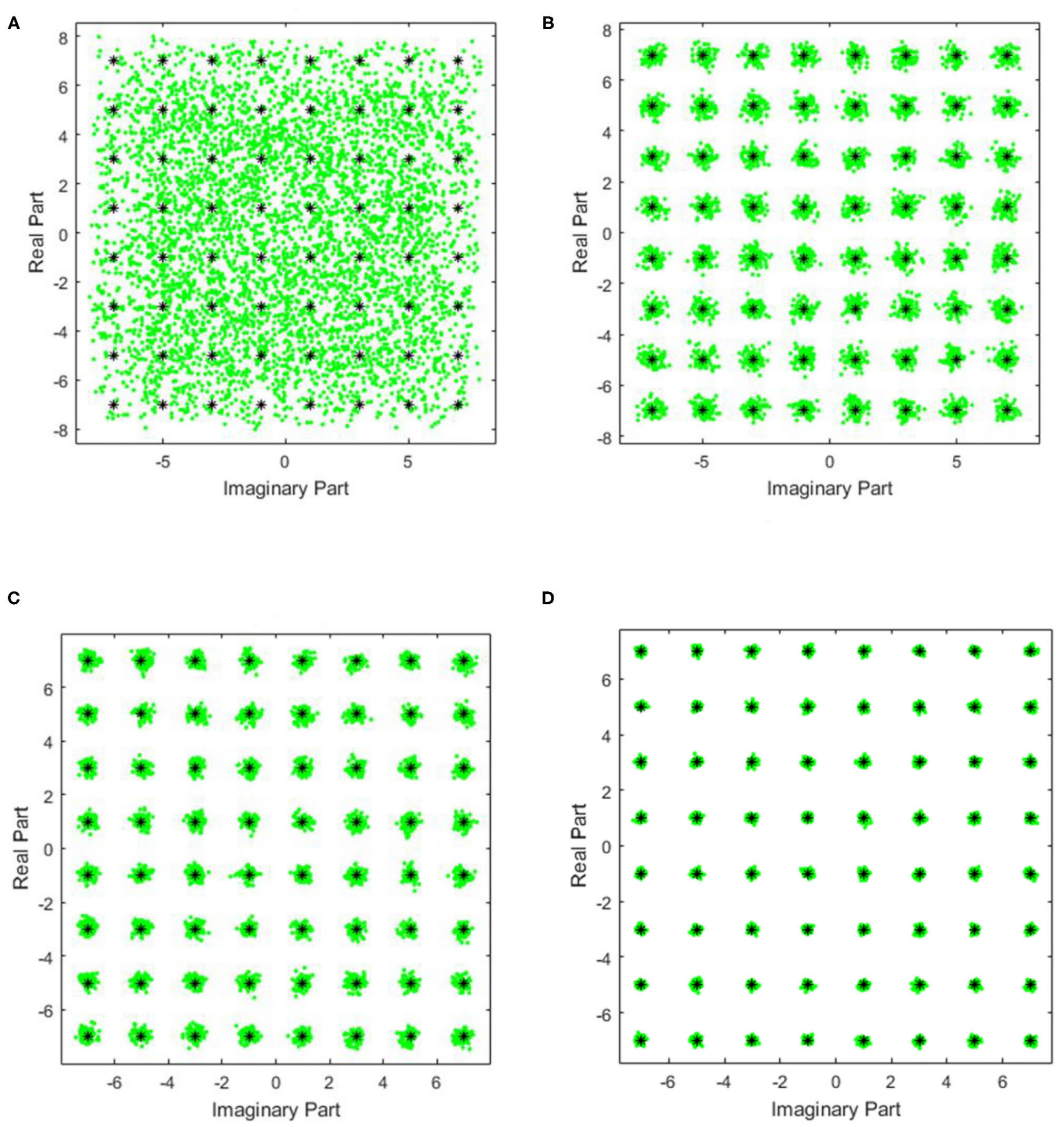
The equalizer input signal diagram and the output constellations of all algorithms. **(A)** The equalizer input signal diagram and the output constellation diagram of **(B)** MCMA, **(C)** Concurrent MCMA-DD, and **(D)** Concurrent MCMA-DD with BB.

Figure 3 shows the equalizer input signal diagram and the output constellation diagrams of the three algorithms. It could be intuitively seen that the proposed concurrent MCMA-DD with BB algorithm compensates the channel distortion more effectively comparing with other algorithms.

**Figure 4:**
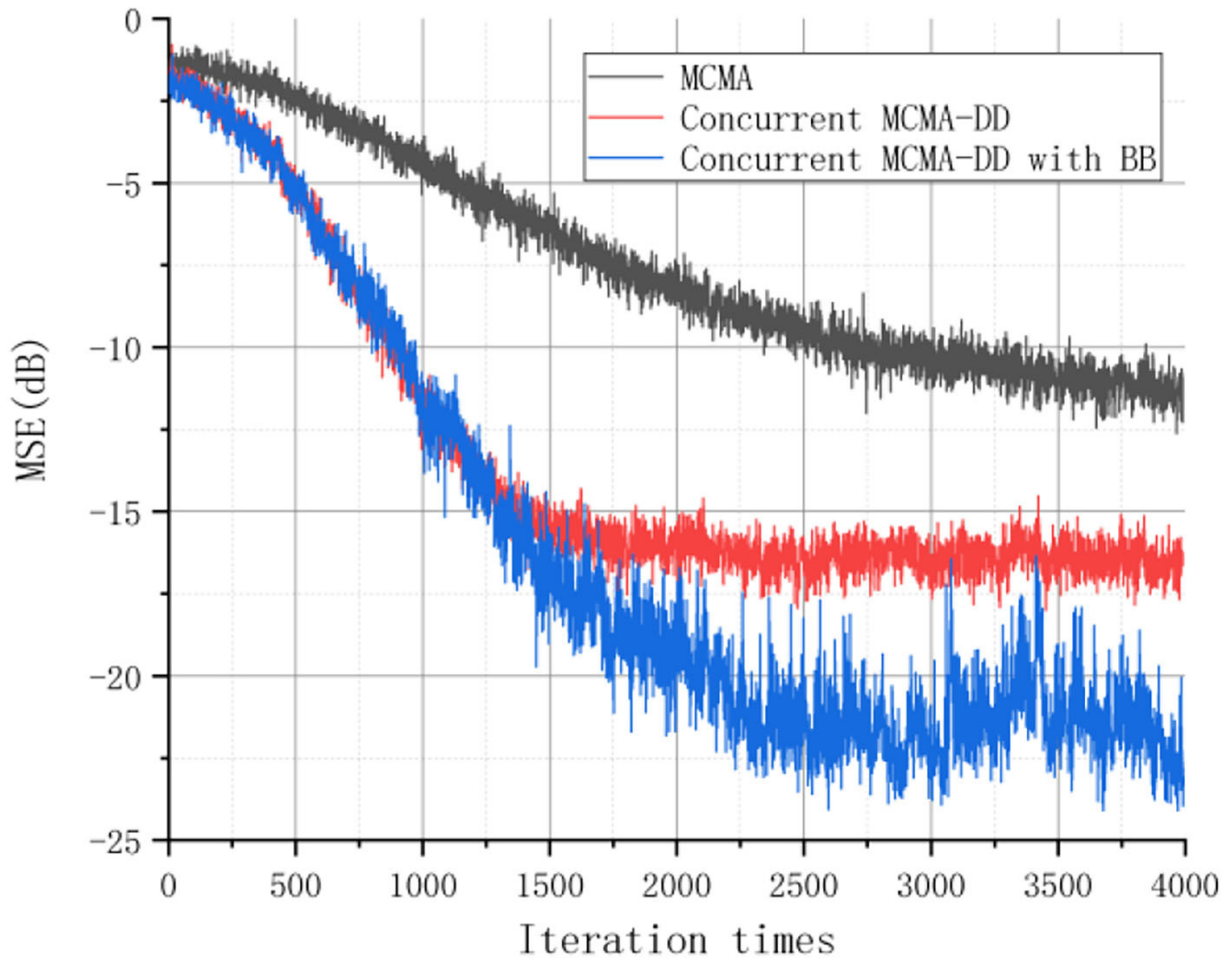
The MSE performance of the three equalizers.

**Figure 5:**
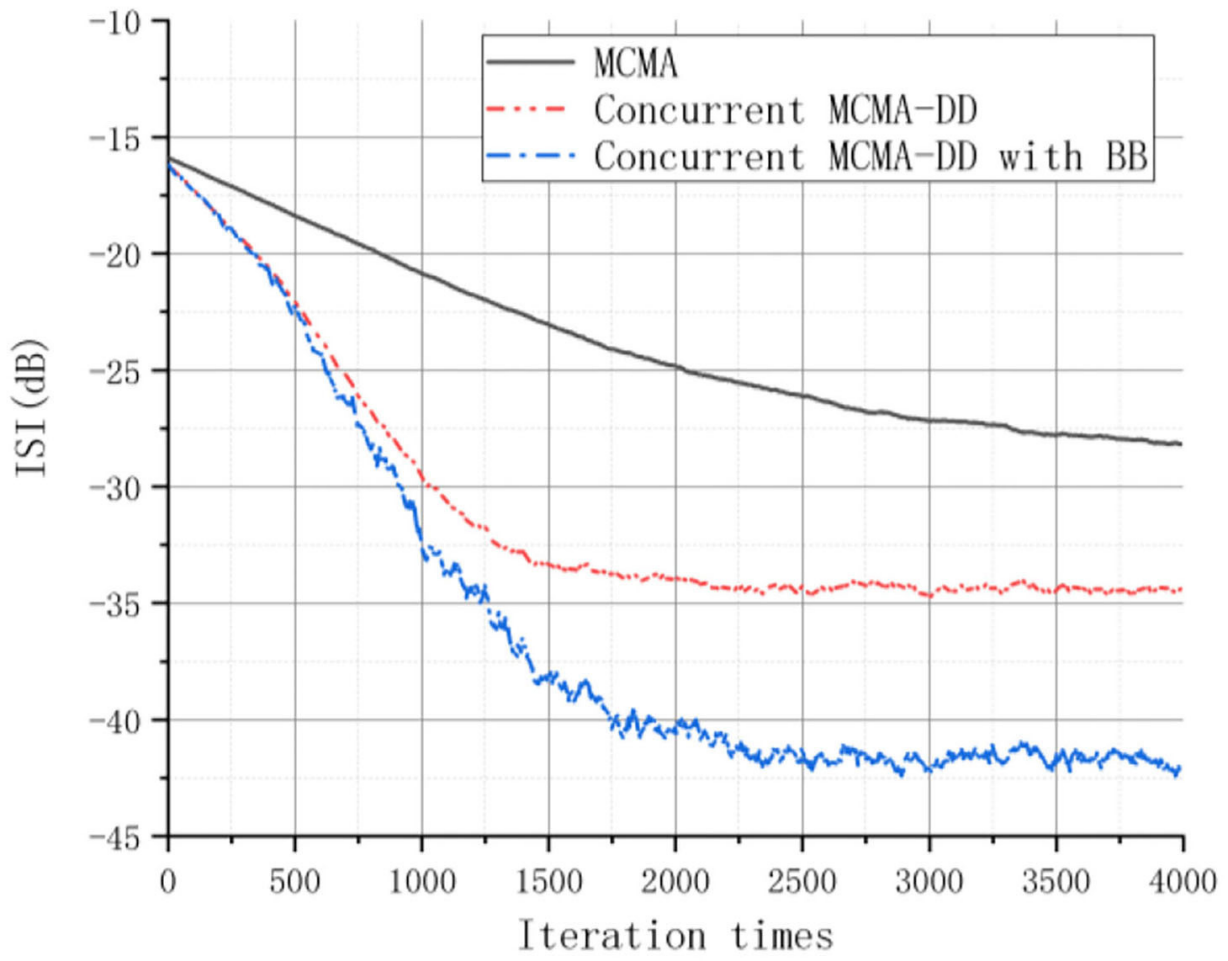
The ISI performance of the three equalizers.

The learning curves for the three equalizers are shown in Figures 4, 5 based on the estimated MSE and ISI measurements. The results indicated that the CS and the SS equalization performance of the concurrent MCMA-DD with BB algorithm is better than the MCMA and the concurrent MCMA-DD algorithm. Specifically, compared to the concurrent MCMA-DD algorithm, the convergence rate of the proposed algorithm was ~100 symbols faster, and the SS error was reduced by 5 dB.

## Conclusions

In this paper, a concurrent MCMA and DD with BB algorithm has been proposed. The proposed algorithm could operate a DD equalizer concurrently with MCMA equalizer and adjust the SS by using BB method. Compared with MCMA and concurrent MCMA-DD, the proposed algorithm exhibits robust equalization performance and rapid CS. The proposed algorithm could be applied to some more complex wireless channel environments, which are of great significance for improving remote control of robot and all wireless communication control system.

There are still some problems to be further studied in this paper:

1. The BB algorithm is applied to the BE algorithm. Although it can reduce SS error and accelerate the CS, the calculation of the algorithm is large, and the complexity is high. Further research is needed to simplify the algorithm and improve its performance.
2. The blind equalization algorithm proposed in this paper also needs to further optimize and analyze the higher-order QAM signal and the more complex digital modulation signal.
3. The proposed blind equalization algorithm is verified by software simulation. On the basis of software simulation, developing and building more practical hardware system is still a subject worthy of further study.

## Data Availability Statement

The original contributions presented in the study are included in the article/supplementary material, further inquiries can be directed to the corresponding author.

## Author Contributions

TX and ZX is the main writer of this paper, proposed the main idea, analyzed the feasibility and of the algorithm, and completed the simulation. HY, YC, JL, and YZ gave some important suggestions for algorithm idea. All authors read and approved the final manuscript.

## Conflict of Interest

HY, YC, JL, and YZ are employed by Guangdong Shenglu Telecommunication Tech. Co., Ltd. The remaining authors declare that the research was conducted in the absence of any commercial or financial relationships that could be construed as a potential conflict of interest.
